# Prevalence of Chronic Obstructive Pulmonary Disease and Asthma in Polycythemia Vera and Essential Thrombocythemia and Its Prognostic Implications

**DOI:** 10.3390/jcm14238416

**Published:** 2025-11-27

**Authors:** Ivan Krecak, Danijela Lekovic, Isidora Arsenovic, Nina Dabcevic, Iva Ivankovic, Hrvoje Holik, Ivan Zekanovic, Martina Moric Peric, Andrea Anic Matic, Andrija Bogdanovic, Marko Skelin, Marko Lucijanic

**Affiliations:** 1 Department of Internal Medicine, General Hospital of Sibenik-Knin County, 22000 Sibenik, Croatia; 2 Faculty of Medicine, University of Rijeka, 51000 Rijeka, Croatia; 3 University of Applied Sciences Sibenik, 22000 Sibenik, Croatia; 4 Clinic of Hematology, University Clinical Center Serbia, 11000 Belgrade, Serbia; 5 Faculty of Medicine, University of Belgrade, 11000 Belgrade, Serbia; 6 Department of Internal Medicine, Dr. Josip Benčević General Hospital, 35000 Slavonski Brod, Croatia; 7 Department of Internal Medicine, General Hospital Zadar, 23000 Zadar, Croatia; 8 Clinic for Pulmonary Disorders, University Hospital Center Split, 21000 Split, Croatia; 9 Pharmacy Department, General Hospital of Sibenik-Knin County, 22000 Sibenik, Croatia; 10 Division of Hematology, University Hospital Dubrava, 10000 Zagreb, Croatia; 11 School of Medicine, University of Zagreb, 10000 Zagreb, Croatia

**Keywords:** COPD, asthma, myeloproliferative neoplasms, thrombosis, survival

## Abstract

**Background:** The prevalence and the prognostic impact of chronic obstructive pulmonary disease (COPD) and asthma in patients with myeloproliferative neoplasms (MPNs) are unknown. **Methods:** This retrospective multicenter cohort analyzed the prevalence and prognostic implications of COPD and asthma in 246 patients with essential thrombocythemia (ET) and polycythemia vera (PV). **Results:** A total of 6.5% and 1.6% patients had COPD or asthma, respectively, without statistically significant differences with respect to disease phenotype. The presence of COPD/asthma was more frequently associated with active/prior smoking (*p* = 0.021) and constitutional symptoms (*p* = 0.001). After the median follow-up of 47.5 months, the presence of COPD/asthma was univariately associated with an inferior time to thrombosis (TTT; median 135 vs. 190 months, 95% confidence interval (CI) 1.8–29.5, hazard ratio-HR 7.75, *p* = 0.005), mainly driven by venous (HR 37.3, 95% CI 3.2–43.6, *p* = 0.003) and not arterial events (HR 1.77, 95% CI 0.40–7.78, *p* = 0.445, *p* = 0.445). Statistically significant interactions existed between COPD/asthma, female sex (HR 3.94, 95% CI 1.01–11.02), ET phenotype (HR 7.1, 95% CI 15.3–16.7), *JAK2* positive status (HR 4.17, 95% CI 1.04–6.9), hydroxyurea use (HR 4.67, 95% CI 1.10–7.43), and the presence of other cardiovascular risk factors (HR 8.1, 95% CI 1.55–10.72) with overall thrombotic risk (interaction *p* < 0.050 for all analyses). Finally, the negative effect of COPD/asthma on TTT persisted in the multivariate analysis (HR 6.54, *p* = 0.010) independently of older (>60 years) age (*p* = 0.030) when being adjusted for other clinically meaningful variables. There was no effect of COPD/asthma on overall survival. **Conclusions**: These results provide an important signal regarding the potentially inferior outcomes in ET/PV patients presenting with these common respiratory disorders and may help to further personalize MPN management.

## 1. Introduction

Polycythemia vera (PV) and essential thrombocythemia (ET) are *BCR::ABL1* negative myeloproliferative neoplasms (MPNs) characterized by overproduction of one or more mature myeloid cell lineages, presence of Janus Kinase 2 (*JAK2*), calreticulin (*CALR*) or myeloproliferative leukemia virus (MPL) gene mutations, chronic inflammatory state, debilitating constitutional symptoms, and high thrombotic risk [1]. Therapeutically, all PV patients receive low-dose aspirin and are phlebotomized with an aim to maintain the hematocrit < 45% as two randomized clinical trials have shown that these interventions may lower cardiovascular risk, whereas ET patients usually receive aspirin if they have previously experienced thrombosis or are *JAK2* positive. High-risk ET and PV patients (those having >60 years or with prior thrombosis) are additionally treated with cytoreduction (hydroxyurea or interferons) [1].

Cardiovascular comorbidities, such as arterial hypertension, hyperlipidemia, and smoking, are frequent in PV and ET patients and may additionally contribute to already high thrombotic risk [2,3]. Therefore, stringent control of cardiovascular risk factors is recommended for all MPN patients. In fact, inadequately controlled cardiovascular risk factors in MPNs may also represent an indication for cytoreductive treatment (i.e., interferons or hydroxyurea) and even twice-daily aspirin in otherwise low-risk patients (those without prior thrombosis and younger than 60 years of age) [4,5,6].

Chronic obstructive pulmonary disease (COPD) and asthma are major public health concerns, and their prevalences are on the rise [7,8,9,10]. These common respiratory disorders significantly affect patients’ quality of life and are associated with an increased cardiovascular risk [11,12]. Besides the traditional cardiovascular risk factors (i.e., smoking) frequently present in patients with COPD and asthma, other pathophysiological mechanisms for the increased thrombotic risk in these patients also exist, such as increased blood hypercoagulability, chronic hypoxemia, pulmonary hypertension, hyperinflation, oxidative stress, and chronic inflammation [11,12].

The current prevalence of COPD and asthma and their prognostic implications in MPN patients are unknown. Additionally, both disorders primarily occur in the elderly, present with chronic inflammation, and are burdened with thrombotic complications. Therefore, the aims of this study were: (1) to analyze the prevalences of COPD andasthma in MPNs, (2) to analyze whether the presence of COPD/asthma in MPNs may be associated with distinct MPN disease features, and (3) to analyze the association of COPD/asthma with thrombotic risk and survival in MPNs.

## 2. Patients and Methods

### 2.1. Study Design and Patient Inclusion Criteria

This multicenter study was conducted at three hospitals in Croatia (General Hospital of Sibenik-Knin County, General Hospital Zadar, and dr. Josip Bencevic Slavonski Brod) and one center in Serbia (Clinical Center Serbia Belgrade) in the period between January 1997 and January 2023 and included PV and ET patients whose disease diagnosis was reassessed for all patients according to 2016 World Health Organization criteria [13]. Clinical and laboratory variables were recorded through electronic medical chart review at the time of MPN disease diagnosis. The presence of COPD/asthma was coded at baseline, that is, if diagnosed by a pulmonologist and recorded as such in the medical documentation at the time of MPN diagnosis. Other cardiovascular risk factors considered were arterial hypertension, hyperlipidemia, and smoking (defined as active/prior vs. never). High-risk PV and ET patients were classified as those with age > 60 years and/or prior (arterial or venous) thrombosis documented as such in the medical records [5]. Constitutional symptoms were defined as the presence of any of the usual MPN-related symptoms: fatigue, night sweats, itching, weight loss (>10% in the preceding 6 months), and fever. Excluded from participation were patients younger than 18 years of age, pregnant women, and those lost to follow-up.

The study was conducted in accordance with the Declaration of Helsinki and was approved by the Ethics Committees from all participating centers; the details are presented at the end of the manuscript.

### 2.2. Statistics

The distribution of data was checked using the Shapiro–Wilk test. One proportion test was used to compare the prevalence of COPD and asthma in ET and PV patients with that in the general population. Categorical variables between the two patient groups were compared with the chi-square or Fisher’s exact test as appropriate, whereas differences in continuous variables were assessed with the Mann–Whitney U test. Univariate and multivariate survival analyses were performed with the Kaplan–Meier method, log-rank test, and Cox regression analysis. Time to thrombosis (TTT) was calculated from the time of diagnosis until the first arterial (myocardial infarction, transitory ischemic attack, or ischemic cerebrovascular stroke, or peripheral arterial occlusion) or venous (hand/leg thrombosis and/or pulmonary embolism). Overall survival (OS) was measured from the time of diagnosis until death or the last follow-up visit. Statistical calculations were performed with MedCalc Statistical software (Ostend, Belgium, version 23.2.7), and a significant *p*-value was set at <0.050 for all presented analyses.

## 3. Results

### 3.1. Patient Characteristics and the Prevalence of COPD and Asthma

A total of 246 patients were included (PV = 154, ET = 92); median age was 68 years (range 20–91), 137 (55.7%) were females, 16 (6.5%) had COPD and 4 (1.6%) had asthma. There were no statistically significant differences in the prevalence of COPD and asthma with respect to disease phenotype (*p* > 0.050 for both analyses). On the other hand, prevalences of COPD (6.5% vs. 10.3%; *p* = 0.051) and asthma (1.6% vs. 6.6%; *p* = 0.001) seemed to be somewhat lower in ET and PV patients than in the general population [7,9]. None of the COPD/asthma patients required long-term oxygen therapy.

Considering the relatively small number of COPD and asthma patients, they were grouped together for the purpose of statistical analyses. Overall patient characteristics and stratified according to the presence of COPD/asthma are shown in Table 1. As presented, patients with COPD/asthma were more frequently active or prior smokers (*p* = 0.021) with constitutional symptoms (*p* = 0.001), whereas there were no differences with respect to other tested clinical and laboratory variables at baseline (*p* > 0.050 for all analyses).

### 3.2. Survival Analyses

The median follow-up time was 47.5 months (range 1–307), and a total of 40 (16.3%) thrombotic events (arterial = 30, venous = 10) occurred during this time. Thrombotic events during follow-up were numerically more frequent in COPD/asthma patients (*n* = 6/20, 30% vs. 34/226, 15%; *p* = 0.083) and were mainly driven by venous events (*n* = 3/20, 15% vs. 7/226, 3.1%; *p* = 0.009) whereas there was no difference in the frequency of arterial events (*n* = 3/20, 15% vs. 27/226, 11.9%; *p* = 0.689).

**Figure 1:**
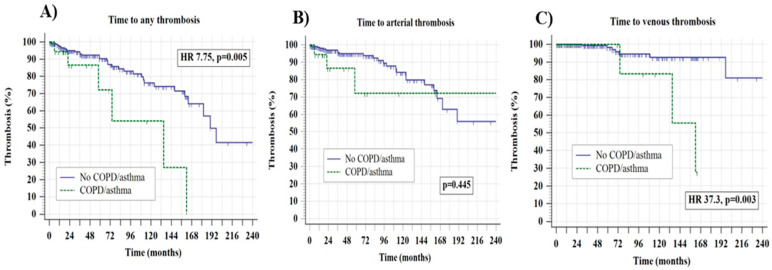
Time to any thrombosis panel (**A**), arterial panel (**B**), and venous thrombosis (**C**) stratified according to the presence of chronic obstructive pulmonary disorder (COPD)/asthma.

In univariate analyses, the presence of COPD/asthma was statistically significantly associated with an inferior TTT in the overall cohort (median 135 vs. 190 months, 95% confidence interval (CI) 1.8–29.5, hazard ratio-HR 7.75 *p* = 0.005) which was also mainly driven by venous (HR 37.3, 95% CI 3.2–43.6, *p* = 0.003) and not arterial events (HR 1.77, 95% CI 0.40–7.78, *p* = 0.445), as shown in Figure 1A–C.

Table 2 summarizes all univariate associations of the clinically most relevant variables with overall, arterial, and venous thrombosis during the follow-up, as well as interactions between these variables and COPD/asthma with overall thrombotic risk. As shown, besides the association of COPD/asthma with overall and venous thrombotic events, other statistically significant associations were found between older (>60 years) age and overall and arterial thrombotic risk, as well as an association between *JAK2* positive status and arterial thrombosis (*p* < 0.050 for all analyses), whereas no other statistically significant associations were recorded. Statistically significant interactions existed between COPD/asthma, female sex, ET phenotype, *JAK2* positive status, hydroxyurea use, and the presence of additional cardiovascular risk factors with thrombotic risk (interaction *p* < 0.050 for all analyses), with a near-significant *p* value for high-risk disease (interaction *p* = 0.063). Targeted analysis of these subgroups with respect to separate arterial vs. venous events was not attempted due to the small number of venous events in specific subsets and the subsequent lack of statistical power.

Finally, in the multivariate Cox regression model, presence of COPD/asthma (HR 6.54, *p* = 0.010) and older (>60 years) age (HR 4.65, *p* = 0.030) were independently of each other associated with an inferior TTT when being additionally adjusted for sex, disease phenotype, prior thrombosis, presence of other cardiovascular risk factors, and the use of aspirin and cytoreduction (*p* > 0.050 for all analyses).

The presence of COPD/asthma did not have an impact on OS in the entire cohort (*p* = 0.310) nor when ET (*p* = 0.412) and PV patients (*p* = 0.575) were analyzed separately.

## 4. Discussion

To our knowledge, this is the first study to investigate the prevalence and the potential prognostic impact of COPD/asthma on major outcomes in ET and PV patients. Several important observations arise from this study. First, patients with COPD/asthma and polycythemia are frequently referred to hematologists for diagnostic exclusion of PV, as these two disorders have been shown to sometimes co-exist [14]. In addition, more recent reports have demonstrated that secondary polycythemia may not be as benign as previously thought, as it may be associated with cardiovascular risk higher than that in the general population and similar to that of low-risk PV [15,16,17]. The presented results suggest that the prevalence of COPD and asthma in MPNs may not be higher than in the general population and was consistent in both time periods analyzed (1997–2009 and 2010–2023). The reasons for this observation remain unknown. Due to a retrospective study design, the potential of selection bias may exist. For example, medical information regarding the presence of these respiratory disorders may not have been captured despite the fact that all documentation was manually reviewed. Also, due to a lack of prospective follow-up, some asymptomatic patients or those with few respiratory symptoms may have been undiagnosed. In addition, some COPD/asthma patients with more severe disease and early deaths before study entry may not have been documented.

On the other hand, some pathophysiological explanations may also exist regarding the lower prevalence of COPD/asthma in MPN patients than in the general population. Besides the bone marrow, thrombocytopoiesis also occurs in the lungs, and platelets are an important source of many cytokines, which possess important immunomodulatory properties and may actually support lung regeneration [18]. This effect has also been shown in patients with MPNs and coronavirus disease 2019 (COVID-19), where patients with higher platelet count actually had improved outcomes [19]. Therefore, it is possible that increased lung thrombocytopoiesis in ET/PV patients may, in fact, help to modulate the inflammatory response in the lungs and thus alleviate respiratory symptomatology, potentially leading to a lower frequency of COPD/asthma. Conversely, one observational cohort study has reported that thrombocytosis in patients with COPD exacerbations may be associated with an inferior one-year survival and reduced in-hospital mortality; cardiovascular hospitalizations were not associated with thrombocytosis, and antiplatelet use improved survival [20]. Therefore, further multinational collaborative studies (preferably prospective), including a larger number of MPN patients, are needed to confirm our findings and to elucidate the exact pathophysiological mechanisms underlying this interesting observation.

Second, it seems that the presence of COPD/asthma does significantly affect MPN disease phenotype, as both patient groups had similar baseline clinical and laboratory characteristics. More specifically, only constitutional symptoms were more frequent in patients with COPD/asthma. The increased frequency of the latter could be, at least partly, attributable to the negative effect of these respiratory disorders on patients’ symptoms and the quality of life. Also, prior or active smoking was more frequent in patients with COPD/asthma. Considering the known widespread detrimental health effects of smoking and the fact that it may also impair treatment responses and survival in MPNs [21], all patients should be strongly advised to quit smoking. Due to a retrospective study design, we were unable to analyze differences in specific symptoms and cytokine profiles between MPN patients with and without COPD/asthma, and further studies should also focus on this topic.

Third, the presence of COPD/asthma was both in univariate and multivariate analyses associated with an inferior TTT, which was mainly driven by venous events. As both COPD and asthma are associated with a hypercoagulable state [11,12,22,23] and an increased risk for both arterial and venous thrombosis in the general population [11,12,24,25,26,27,28,29], a significant prothrombotic synergism between COPD/asthma and MPN may indeed exist. However, in the current study, the presence of COPD/asthma was associated with an increased risk of venous, but not arterial, thrombosis. A similar effect has also been shown in MPN patients with COVID-19, where a significant risk of venous thromboembolism has been noted [19]. In addition, a significant proportion of study patients used aspirin as thromboprophylaxis per the current MPN guidelines, and hydroxyurea, which are not that potent in the protection of the venous district [30]. Also, patients with COPD/asthma are often less physically active and more sedentary, leading to venous stasis, which predisposes them more to venous than arterial thrombosis. Moreover, there were statistically significant interactions between female sex, ET phenotype, hydroxyurea use, *JAK2* positivity, high-risk disease, and the presence of other cardiovascular risk factors with overall thrombotic risk, suggesting that special consideration and clinical surveillance should be targeted at this specific MPN patient subpopulation having these high-risk features [1,4]. It should also be pointed out that both hydroxyurea use and high-risk may present with overlapping prognostic properties, as both may represent the same patient subpopulation. Finally, it should be acknowledged that due to the aforementioned competing-risk caveats, a small number of study patients and events leading to overfitting and wide CI, some of our analyses may lack sufficient power. Therefore, additional studies on a larger number of MPN patients are needed to confirm our observations.

It should also be noted that both COPD/asthma and MPN patients during the disease course may also eventually develop pulmonary hypertension, which is associated with inferior outcomes in MPNs [31,32]. Collectively, the presented results suggest that careful clinical surveillance may be needed for MPN patients with COPD and asthma, with stringent control of other cardiovascular risk factors. Prospective cohort studies on a larger number of MPN patients with COPD/asthma are needed to unravel optimal treatment approaches for this specific patient population.

Limitations of this study are its retrospective design, the small number of COPD and asthma MPN patients included, and the small number of events of interest during follow-up. Additionally, due to the retrospective design of the study, we could not assess other important variables of interest in MPN patients with COPD/asthma, such as the effect of pulmonary function tests, pulmonary arterial pressure, and other echocardiographic findings on different clinical outcomes. Also, the potential effect of the number and the severity of COPD/asthma exacerbations on ET/PV disease outcomes was not assessed—the negative effect of COPD exacerbations on cardiovascular risk has been demonstrated in the general population [33,34].

Nevertheless, the presented study provides an important signal regarding the potentially inferior clinical outcomes of ET and PV patients with COPD/asthma and may help to further personalize MPN management. Additional prospective cohort studies on a larger number of MPN patients with COPD/asthma using standardized respiratory phenotyping are needed, additionally including patients with primary and secondary myelofibrosis, to validate these results and to elucidate optimal treatment approaches for this MPN patient population.

## Figures and Tables

**Table 1:** Overall patient characteristics and stratified according to COPD/asthma presence.

Variable	Overall (*n* = 246)	Non COPD-Asthma (*n* = 226, 91.6%)	COPD/Asthma (*n* = 20, 8.1%)	*p* Value *
Sex, female	137 (55.7%)	127 (56.2%)	10 (50%)	0.593
Age, years (median, range)	68 (20–91)	68 (20–91)	68.5 (53.84)	0.379
Year of MPN diagnosis				0.596
1997–2009	48 (19.5%)	45 (93.7%)	3 (6.2%)	
2010–2023	198 (80.5%)	181 (91.4%)	17 (8.6%)	
PV ET	154 (62.6%) 92(37.4%)	140 (61.9%) 86 (38.1%)	14 (70%) 6 (30%)	0.476
Palpable splenomegaly	49 (19.9%)	45 (19.9%)	4 (20%)	0.992
Constitutional symptoms	128 (52%)	110 (48.7%)	18 (90%)	**0.001**
JAK2-V617F Calreticulin	189 (76.8%) 16 (6.5%)	171 (75.7%) 15 (6.6%)	18 (90%) 1 (5%)	0.146 0.708
History of thrombosis	63 (25.6%)	58 (25.7%)	5 (25%)	0.948
High-risk disease **	196 (79.7%)	181 (80.1%)	15 (75%)	0.588
Hydroxyurea	170 (69.1%)	156 (69%)	14 (70%)	0.928
Aspirin	201 (81.7%)	186 (82.3%)	15 (75%)	0.419
Warfarin	38 (15.4%)	34 (15%)	4 (20%)	0.557
Arterial hypertension	184 (74.8%)	169 (74.8%)	15 (75%)	0.982
Hyperlipidemia	92 (37.4%)	85 (37.6%)	7 (35%)	0.817
Smoking (current/previous vs. never)	58 (23.6%)	50 (22.1%)	9 (45%)	**0.021**
Total leukocytes, ×10^9^/L (median, range)	9.6 (1.5–26.2)	9.5 (1.5–26.2)	10.4 (4.5–20.7)	0.163
Granulocytes, ×10^9^/L (median, range)	7.1 (0.34–20.6)	6.69(0.34–20.6)	7.64 (4.82–17.78)	0.278
Lymphocytes. ×10^9^/L (median, range)	2.01 (0.15–5.47)	1.97 (0.15–5.02)	2.2 (1.41–5.47)	0.062
Erythrocytes, ×10^12^/L (median, range)	5.47 (1.56–21)	5.49 (1.56–21)	5.54 (2.9–9.5)	0.804
Platelets, ×10^9^/L (median, range)	570 (104–3211)	570 (104–3211)	595 (261–1055)	0.673
Hemoglobin, g/L (median, range)	157 (148–229)	157 (148–229)	159 (115–195)	0.855
Hematocrit, % (median, range)	48.3 (34–90)	48.3 (34–90)	50.2 (37.9–62)	0.534

* Statistically significant *p* values are bolded and set at <0.050. The chi-square, Fisher’s exact, and the Mann–Whitney U test were used. ** High-risk disease = age > 60 years of age or prior thrombosis. COPD = chronic obstructive pulmonary disorder, ET = essential thrombocythemia, PV = polycythemia vera.

**Table 2:** Associations of the most clinically relevant variables with overall, arterial, and venous thrombosis and the interaction analysis of these variables with chronic obstructive pulmonary disease (COPD/asthma) and overall thrombotic risk.

Variable	Overall Thrombosis (*n* = 40)	Arterial Thrombosis (*n* = 30)	Venous Thrombosis (*n* = 10)	Interaction Between COPD/Asthma and Overall Thrombotic Risk
COPD/asthma	**HR 7.75, 95 CI 1.8–29.5, *p* = 0.005**	HR 1.77, 95% CI 0.40–7.78, *p* = 0.445	**HR 37.3, 95% CI 3.2–43.6, *p* = 0.003**	**-**
Female sex	HR 1.03, 95% CI 0.55–1.94 *p* = 0.903	HR 1.64, 95% CI 0.76–3.52, *p* = 0.201	HR 0.41, 95% CI 0.11–1.45, *p* = 0.169	**HR 3.94, 95% CI 1.01–11.02, interaction *p* = 0.047**
Age > 60 years	**HR 2.15, 95% CI 1.1–4.2, *p* = 0.026**	**HR 2.83, 95% CI 1.25–6.4, *p* = 0.012**	HR 0.93, 95% CI 0.24–3.59, *p* = 0.927	HR 2.9, 95% CI 0.85–9.44, interaction *p* = 0.088
ET phenotype	HR 1.08, 95% CI 0.56–2.07, *p* = 0.805	HR 1.55, 95% CI 0.70–3.41, *p* = 0.275	HR 0.66, 95% CI 0.18–2.85, *p* = 0.529	**HR 7.1, 95% CI 15.3–16.7, interaction *p* = 0.007**
JAK2 vs. other/negative mutations	HR 1.68, 95% CI 0.82–3.44, *p* = 0.153	**HR 2.50, 95% CI 1.1–5.9, *p* = 0.037**	HR 1.62, 95% CI 0.39, 6.7, *p* = 0.503	**HR 4.17, 95% CI 1.04–6.9, interaction *p* = 0.041**
History of thrombosis	HR 0.72, 95% CI 0.32–1.58, *p* = 0.419	HR 0.80, 95% CI 0.30–2.13, *p* = 0.665	HR 0.39, 95% CI 0.07–2.1, *p* = 0.278	HR 1.48, 95% CI 0.58–10.1, interaction *p* = 0.223
High-risk disease *	HR 1.83, 95% CI 0.92–3.72 *p* = 0.092	HR 1.55, 95% CI 0.65–3.68, *p* = 0.315	HR 3.01, 95% CI 0.73–12.4, *p* = 0.126	HR 3.43, 95% CI 0.94–7.63, interaction *p* = 0.063
Hydroxyurea	HR 1.43, 95% CI 0.69–2.9, *p* = 0.339	HR 1.07, 95% CI 0.44–2.62, *p* = 0.870	No events	**HR 4.67, 95% CI 1.10–7.43, interaction *p* = 0.030**
Aspirin	HR 0.67, 95% CI 0.32–1.42, *p* = 0.302	HR 0.68, 95% CI 0.27–1.69, *p* = 0.411	HR 0.93, 95% CI 0.23–3,70, *p* = 0.922	HR 0.04, 95% CI 0.29–4.8, interaction *p* = 0.827
Cardiovascular risk factors **	HR 0.78, 95% CI 0.35–1.73, *p* = 0.554	HR 0.70, 95% CI 0.28–1.75, *p* = 0.453	HR 0.72, 95% CI 1.6–3.1, *p* = 0.663	**HR 8.1, 95% CI 1.55–10.72, interaction *p* = 0.004**

Statistically significant results are bolded. The Kaplan–Meier, the log-rank test and the interaction analysis were used. * High-risk disease (age > 60 years of age and/or prior thrombosis), ** Cardiovascular risk factors included the presence of arterial hypertension, hyperlipidemia, or active/prior smoking. HR = hazard ratio, CI = confidence interval, COPD = chronic obstructive pulmonary ET = essential thrombocythemia, JAK2 = Janus Kinase 2.

## Data Availability

The data presented in this study are available on request from the corresponding author (Ivan Krecak) due to privacy reasons.
